# Clinical effects of probiotic or azithromycin as an adjunct to scaling and root planning in the treatment of stage III periodontitis: a pilot randomized controlled clinical trial

**DOI:** 10.1186/s12903-020-01276-3

**Published:** 2021-01-07

**Authors:** Alicia Morales, Rafael Contador, Joel Bravo, Paola Carvajal, Nora Silva, Franz-Josef Strauss, Jorge Gamonal

**Affiliations:** 1 Department of Conservative Dentistry, Faculty of Dentistry, University of Chile, Avenida Sergio Livingstone 943, Comuna de Independencia, Santiago, Chile; 2 Centro de Epidemiología Y Vigilancia de Las Enfermedades Orales (CEVEO), Faculty of Dentistry, University of Chile, Santiago, Chile; 3 University of Antofagasta, Antofagasta, Chile; 4 Laboratory of Microbiology, Department of Medicine and Oral Pathology, Faculty of Dentistry, University of Chile, Santiago, Chile; 5 Department of Oral Biology, Medical University of Vienna, Vienna, Austria; 6 Clinic of Reconstructive Dentistry, Center of Dental Medicine, University of Zurich, Zurich, Switzerland

**Keywords:** Periodontitis, Scaling and root planing, *Lactobacillus rhamnosus*, Azithromycin

## Abstract

**Background:**

The aim of this triple-blind placebo-controlled parallel-arm randomized clinical trial was to evaluate the clinical effects of *Lactobacillus rhamnosus* SP1 or azithromycin as an adjunct to scaling and root planing (SRP) in patients with stage III periodontitis.

**Methods:**

Forty-seven systemically healthy participants with stage III periodontitis were recruited. Following SRP, the participants were randomly assigned to one of three treatment modalities; (1) placebo (n = 15), (2) probiotics (n = 16) and (3) antibiotics-azithromycin (n = 16). The participants were monitored at baseline, 3, 6, 9 and 12 months after therapy. Probing pocket depth (PPD), bleeding on probing (BOP), clinical attachment loss (CAL) and plaque accumulation (PI) were evaluated.

**Results:**

All 47 participants completed the study. At 12 months, all groups showed significant improvements of PPD and PI (*p* < 0.012) irrespective of the treatment modality and without significant differences between the groups. Probiotics and azithromycin showed no added benefit in terms of CAL. While the placebo (*p* = 0.002) and the antibiotic-azithromycin (*p* = 0.002) group showed a significant reduction of BOP, only the placebo group revealed a significant reduction of CAL at 12 months follow-up (*p* = 0.003). The number of sites and teeth with PPD ≥ 5, ≥ 6 and ≥ 7 mm were significantly reduced in all groups at 12 months follow-up (*p* < 0.025) irrespective of the treatment regime and without significant differences between the groups.

**Conclusion:**

The use of probiotics or azithromycin as an adjunct to SRP failed to provide additional benefits in the treatment of stage III periodontitis. The benefits of these two treatment regimes as an adjunct to SRP remain unclear.

**Trial registration:**

NCT02839408, 10/28/2017, Clinicaltrial.gov.

## Background

Periodontitis is characterized by a microbially‐associated and host‐mediated inflammation resulting in the loss of periodontal attachment [1]. This is caused by a dysbiotic microbiome in the subgingival biofilm in a susceptible host. The gold standard treatment to manage periodontitis is scaling and root planing (SRP) [2, 3]. This treatment allows the removal of supra- and subgingival deposits, cementum or surface dentin that is rough, impregnated with calculus or contaminated with toxins or microorganisms [4]. However, SRP may also fail. This failure could be due to the presence of deep pockets and the ensuing difficulties during the instrumentation, including the inaccessibility of certain areas such as furcations [5]. Therefore, patients with deep pockets, progressive or ‘active’ disease, or specific microbiological profile, can benefit from an antibiotic adjunctive therapy [6].

Systemic antibiotics have the advantage of reaching all oral surfaces and fluids, in addition to having the potential to reach periodontal pathogens that eventually invade the host’s tissue [7]. Azithromycin is a broad-spectrum bacteriostatic antibiotic [8] with immunomodulatory properties [9]. Due to its pharmacological properties azithromycin allows a once a day administration over 3 or 5 days, thus increasing patient compliance [8] and limiting the side effects. Azithromycin as an adjunct to SRP significantly improves the efficacy of non-surgical periodontal therapy in terms of probing pocket depth (PPD) and bleeding on probing (BOP) reduction along with clinical attachment level (CAL) gain [5, 7]. SRP in conjunction with antibiotics is nevertheless not always associated with superior clinical results. The frequent recolonization of treated sites by periodontopathogens, as well as microbial resistance emergency calls for new therapeutic approaches for managing periodontitis. One such approach is the use of probiotics [9].

Probiotics are “living microorganisms, mainly bacteria, that are safe for human consumption and when taken in proper quantities may provide beneficial effects for the human health” [10]. The modes of action of probiotics are related to their ability to enhance mucosal barrier, produce antimicrobial agents, compete and exclude pathogenic agents, and modulate immune response. *Lactobacillus* and *Bifidobacterium* are the most common genera of bacteria in probiotics [11, 12]. *Lactobacillus rhamnosus* SP1 also known as *L. rhamnosus* GG, has been proposed as an adjunct to SRP, thanks to its ability to inhibit the growth of periodontopathogens through bacteriocins [13, 14], its resistance to environmental stress [15], its immunomodulatory effect [16–18] and its inability to influence the acidogenicity of the supragingival plaque [19]. However, the available evidence regarding the clinical effects of probiotics as an adjunct to scaling and root planing (SRP) is controversial. While some systematic reviews and narrative reviews [20] claim improved clinical outcomes, a recent systematic review concluded that probiotics do not provide clear clinical benefits [21]. Thus, it remains unclear whether probiotics have an added benefit to SRP, thereby preventing their wide use in clinical practice.

The aim of the present study was, therefore, to evaluate the clinical effect of *L. rhamnosus* SP1 or azithromycin in the treatment of patients with stage III periodontitis. The null hypothesis states that either probiotics or azithromycin as an adjunct to SRP in stage III periodontitis, do not lead to superior clinical outcomes compared to those obtained with SRP alone.

## Methods

### Ethical guidelines

This trial started in June 2014 and finished in August 2016. This triple-blind placebo-controlled parallel-arm randomized clinical trial was performed following guidelines recognized by the Declaration of Helsinki, as revised in 2013 for experimentation involving human participants. The study was approved by the Local Ethical Committee of the Faculty of Dentistry at the University of Chile (Decision number: 2012/08). The present report adheres to Consolidated Standards of Reporting Trials (CONSORT) guidelines and was registered in clinicaltrial.gov (identifier no. NCT02839408). The protocol of the study was explained to all patients, and informed consent was obtained after explanation of the purpose, nature, risks and benefits of participating in this study. The study details have been reported elsewhere [22].

### Patient selection criteria

Ninety-six volunteers were initially examined in the Faculty of Dentistry, University of Chile, of which 47 were included in the present study. Eligibility criteria for participants were as follows: (a) adult patients—aged 35 or older; (b) self-reported ‘good health’, with an absence of a medical history of a chronic disease or taking medication known to affect periodontal disease; (c) non-institutionalized male or female; (d) presence of a minimum of 14 teeth (excluding third molars); (d) presence of at least 10 posterior teeth (e) Presence of at least 5 teeth with periodontal sites with PPD ≥ 5 mm and clinical attachment loss (CAL) ≥ 3 mm, BOP ≥ 20% and extensive radiographically determined bone loss.

The exclusion criteria were as follows: (a) pregnancy or lactation; (b) having received any periodontal treatment in the 6-month period before the study; (c) having received non-steroidal anti-inflammatory medication, antibiotics or probiotics therapy in the past 6 months (prior to study).

### Randomization of the study participants

**Fig. 1:**
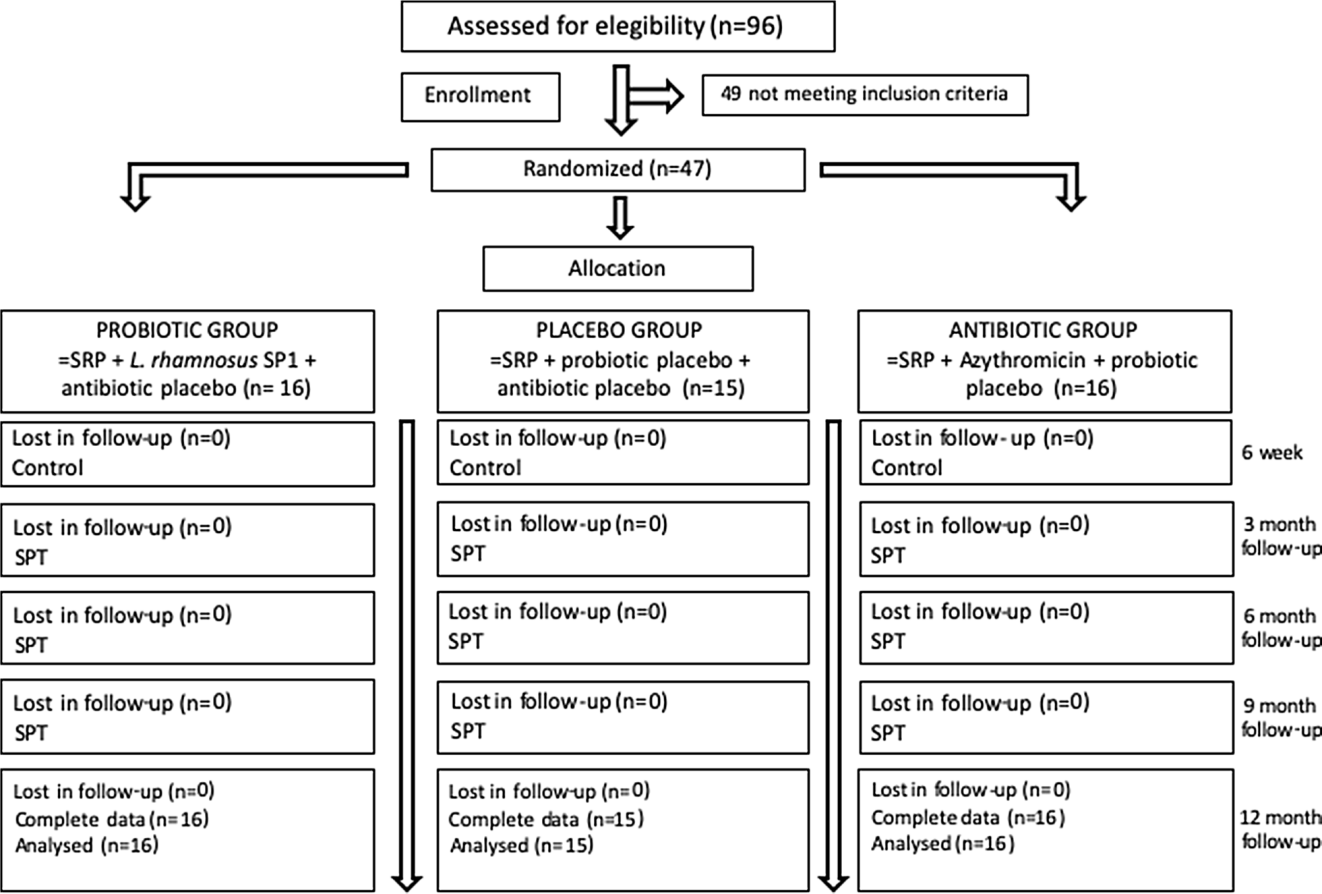
Flow chart of the study design

Eligible individuals were randomly (simple randomization) allocated to groups according to gender, age, and smoking status after the basal examination using a computer-generated list. All participants were enrolled to one of the three treatment groups: (1) placebo (SRP + azithromycin placebo + probiotic placebo), (2) probiotic (SRP + probiotic + azithromycin placebo) or antibiotic (SRP + azithromycin + probiotic placebo) group (Fig. 1). Concealed allocation was performed using opaque, sealed envelopes containing probiotics or antibiotics arranged by an appointed research assistant. For the purpose of maintaining full blinding throughout the study period, the randomization code and details of the study groups were held by a research assistant only from the day of recruitment and were not revealed until all data had been collected and analyzed.

### Clinical assessment

Periodontal clinical parameters including probing pocket depth (PPD), bleeding on probing (BOP), clinical attachment loss (CAL) and plaque index (PI) were evaluated at six sites in all teeth, excluding third molars using a manual probe (UNC probe; Hu-Friedy Mfg. Co. Inc., Chicago IL, USA). All measurements were conducted by one calibrated examiner (AM). Clinical examinations were performed at baseline, 3, 6, 9 and 12 months after treatment.

### Periodontal therapy

After baseline examinations, all patients received non-surgical periodontal therapy. Scaling and root planing (SRP) per quadrant (4–6 sessions, by PC, RC and NS) were performed using an ultrasonic scaler (Cavitron, Dentsply, York, PA, USA) and Gracey curettes (Hu Friedy Mfg. Co. Inc., Chicago, IL, USA). Treatment included oral hygiene instructions, using a manual toothbrush. The patients started taking the placebo, probiotic or antibiotics after the last session of SRP. The Placebo group received probiotic placebo sachets and antibiotic placebo capsules. Probiotic group received probiotic sachets and antibiotic placebo capsules. Antibiotic group received antibiotic capsules and probiotic placebo sachets. Identical sachets were presented to patients. Individuals were instructed to dissolve 1 sachet (*Lactobacillus rhamnosus* SP1 (2 × 10^7^ colony forming units/day) (Macrofood S.A., Santiago, Chile) or probiotic placebo sachets in 150 ml of water and ingest it once a day after brushing their teeth, for 3 months. Azithromycin 500 mg (capsules) or antibiotic placebo capsules were ingested once a day for 5 days. Placebo sachets (probiotic placebo) and placebo capsules (antibiotic placebo) were identical in taste, texture, and appearance to the probiotic sachets and antibiotic capsules. Periodontal supportive therapy was performed every 3 months (by PC). The therapy provided at the maintenance appointment included removal of plaque and calculus, utilizing curets, ultrasonic devices, and rubber cup low‐speed polish as suggested by the American Academy of Periodontology position paper [23]. Behavior modification (tobacco cessation, oral hygiene instruction, and systemic factor counseling) was performed based on patient findings.

### Compliance and adverse reactions

All patients returned capsules with antibiotics or placebo at 6-week visits and probiotics or placebo sachets at 1, 2 and 3-month visits. At every visit, patients received new sachets. In order to check the patient’s compliance, they were called by phone every week. In each control visit or phone call, the clinical examiner (AM) inquired after general health changes, use of mouth rinses, use of probiotic products and any adverse events.

### Outcome variables

The primary outcome was the change in CAL. Secondary outcome variables were changes in PPD, PI and BOP, percentages of patients, teeth and sites with PPD ≥ 5 mm, ≥ 6 mm, ≥ 7 mm. Sub-analyses were performed on CAL and PPD, taking into account the initial PPD. A pocket was considered moderate if its initial PPD was between 4 and 6 mm and deep if ≥ 7 mm. Changes or delta (∆) in clinical parameters (at subject level) from baseline to 3, 6, 9 and 12 months were determined. “Pocket” closure was defined as mean and SD of percentage of sites going from PPD ≥ 4 mm to PPD ≤ 3 mm at 3- and 12-months follow up [7]. “Risk for disease progression” was defined at the patient level according to Lang and Tonetti [24]. Low risk was defined as ≤ 4 sites with PPD ≥ 5 mm, moderate risk was defined as 5–8 sites with PD ≥ 5 mm, and high risk was defined as ≥ 9 sites with PD ≥ 5 mm [24]. The “need for additional periodontal treatment” was defined as persisting pockets ≥ 5 mm with BOP [25].

### Statistical analysis

Sample size calculation was performed using CAL as the primary outcome variable. A significance level of α = 5% α and a power level of 80% was defined. Considering a difference ≥ 1 mm between groups in CAL changes and a standard deviation of 0.8 mm [26], 14 participants per group were necessary to detect potential differences. For all statistical evaluations, the patient was the unit of measurement. The Shapiro–Wilk test was used to test the normality of the data sets. Quantitative data were recorded as the mean value ± standard deviation (SD) or percentage (%). The inter-group differences were determined using Fisher’s exact test, Kruskall Wallis test and ANOVA depending on the distribution of the data. Intra-group differences in clinical parameters over time were determined by the Related Samples Friedman's test (*p* < 0.05) and. The Bonferroni-corrected Wilcoxon signed-rank test and Bonferroni-corrected Mc Nemar test were used to evaluate the intragroup multiple comparisons (*p* < 0.0125 or *p* < 0.025). Effect Sizes (‘mean change’ divided by ‘standard deviation of the baseline values’) were calculated based on changes in clinical parameters from baseline to 3, 6, 9 and 12 months or from baseline to 3 and 12 months follow-up. The statistical analysis was performed using a statistical package (StataCorp, College Station, TX, USA).

## Results

Forty-seven patients were analyzed; 16 in the probiotic group, 16 in the antibiotic group and 15 in the placebo group. All participants completed the study period and complied with the study requirements (Fig. 1). Adverse events were observed in one participant from the antibiotic group (nausea). The periodontal diagnosis of all participants was Stage III Periodontitis generalized grade B. Demographic, medical and clinical characteristics are described in Table 1. No significant differences were found between groups at baseline (*p* > 0.05).

**Table 1:** Baseline data of patients in the treatment groups, intra- and inter-group comparisons of clinical parameters at baseline, 3-, 6-, 9- and 12-months follow up

	Baseline (a)	3 months (b)	6 months (c)	9 months (d)	12 months (e)	*p* value (intra-group)	Post-hoc (Bonferroni adjustment)	Effect size (∆3 months-BL)^f^	Effect size (∆6 months-BL)^g^	Effect size (∆9 months-BL)^h^	Effect size (∆12 months-BL)^i^	∆3 months-BL	∆6 months-BL	∆9 months-BL	∆12 months-BL
*Age (years)*															
Probiotic group (n = 16)	46.5 ± 9.3														
Antibiotic group (n = 16)	49.0 ± 7.9														
Placebo group (n = 15)	52.8 ± 7.5														
*p* value (inter-group)	0.1171														
*Gender (M/F) (%)*															
Probiotic group (n = 16)	50.0 / 50.0														
Antibiotic group (n = 16)	63.5 / 37.5														
Placebo group (n = 15)	53.3 / 46.7														
*p* value (inter-group)	0.8150														
*Smokers (%)*															
Probiotic group (n = 16)	43.8														
Antibiotic group (n = 16)	18.8														
Placebo group (n = 15)	40.0														
*p* value (inter-group)	0.3440														
*CAL (mm)*															
Probiotic group (n = 16)	3.8 ± 0.7	3.4 ± 0.6	3.5 ± 0.6	3.5 ± 0.7	3.7 ± 0.6	0.0001	**a > b (p = 0.0013)**	− 0.4 ± 0.3	− 0.3 ± 0.4	− 0.3 ± 0.3	− 0.1 ± 0.3	− 0.4 ± 0.4	− 0.3 ± 0.4	− 0.3 ± 0.4	− 0.1 ± 0.3
							a > c (p = 0.0151)								
							**a > d (p = 0.0072)**								
							a > e (p = 0.3520)								
Antibiotic group (n = 16)	4.4 ± 0.9	3.8 ± 0.8	4.0 ± 1.0	4.1 ± 1.0	4.1 ± 1.1	0.0001	**a > b (p = 0.0013)**	− 0.4 ± 0.3	− 0.4 ± 0.4	− 0.3 ± 0.4	− 0.3 ± 0.4	− 0.5 ± 0.4	− 0.4 ± 0.4	− 0.3 ± 0.4	− 0.3 ± 0.5
							**a > c (p = 0.0013)**								
							a > d (p = 0.0262)								
							a > e (p = 0.0262)								
Placebo group (n = 15)	4.7 ± 1.5	4.1 ± 1.4	4.2 ± 1.4	4.3 ± 1.5	4.4 ± 1.5	0.0001	**a > b (p = 0.0013)**	− 0.6 ± 0.4	− 0.5 ± 0.4	− 0.4 ± 0.4	− 0.4 ± 0.3	− 0.6 ± 0.5	− 0.6 ± 0.4	− 0.4 ± 0.5	− 0.4 ± 0.4
							**a > c (p = 0.0019)**								
							**a > d (p = 0.0092)**								
							**a > e (p = 0.0031)**								
*p* value (inter-group)	0.0823	0.2821	0.3254	0.1589	0.4271							0.2628	0.2906	0.6268	0.1217
*PPD (mm)*															
Probiotic group (n = 16)	2.7 ± 0.6	2.2 ± 0.4	2.3 ± 0.4	2.2 ± 0.3	2.3 ± 0.3	0.0001	**a > b (p = 0.0007)**	− 0.2 ± 0.1	− 0.2 ± 0.2	− 0.2 ± 0.1	− 0.1 ± 0.2	− 0.5 ± 0.4	− 0.5 ± 0.5	− 0.5 ± 0.4	− 0.4 ± 0.5
							**a > c (p = 0.0032)**								
							**a > d (p = 0.0008)**								
							**a > e (p = 0.0061)**								
Antibiotic group (n = 16)	2.9 ± 0.4	2.2 ± 0.3	2.3 ± 0.3	2.3 ± 0.3	2.3 ± 0.3	0.0001	**a > b (p = 0.0006)**	− 0.2 ± 0.2	− 0.2 ± 0.1	− 0.2 ± 0.1	− 0.2 ± 0.1	− 0.7 ± 0.5	− 0.6 ± 0.3	− 0.6 ± 0.3	− 0.6 ± 0.4
							**a > c (p = 0.0004)**								
							**a > d (p = 0.0004)**								
							**a > e (p = 0.0008)**								
Placebo group (n = 15)	3.2 ± 0.9	2.4 ± 0.5	2.4 ± 0.5	2.5 ± 0.6	2.4 ± 0.55	0.0001	**a > b (p = 0.0007)**	− 0.3 ± 0.2	− 0.3 ± 0.2	− 0.2 ± 0.2	− 0.3 ± 0.2	− 0.8 ± 0.5	− 0.8 ± 0.6	− 0.7 ± 0.6	− 0.7 ± 0.5
							**a > c (p = 0.0030)**								
							**a > d (p = 0.0023)**								
							**a > e (p = 0.0008)**								
*p* value (inter-group)	0.2436	0.5916	0.4425	0.5391	0.8799							0.2774	0.2266	0.4099	0.1511
*BOP (%)*															
Probiotic group (n = 16)	49.3 ± 18.1	39.2 ± 14.9	42.1 ± 13.6	42.4 ± 14.6	43.0 ± 12.3	0.0001	a > b (p = 0.0386)	− 0.2 ± 0.3	− 0.1 ± 0.4	− 0.1 ± 0.3	− 0.1 ± 0.2	− 10.0 ± 16.6	− 7.1 ± 19.1	− 6.9 ± 13.5	− 6.3 ± 11.2
							a > c (p = 0.1961)								
							a > d (p = 0.0703)								
							a > e (p = 0.0494)								
Antibiotic group (n = 16)	57.4 ± 10.2	41.9 ± 10.9	44.1 ± 13.5	48.1 ± 14.1	42.3 ± 13.3	0.0005	**a > b (p = 0.0022)**	− 0.3 ± 0.2	− 0.2 ± 0.3	− 0.2 ± 0.3	− 0.3 ± 0.2	− 14.7 ± 11.1	− 13.2 ± 14.9	− 9.2 ± 15.0	− 15.0 ± 12.6
							**a > c (p = 0.0052)**								
							a > d (p = 0.0437)								
							**a > e (p = 0.0023)**								
Placebo group (n = 15)	52.5 ± 12.6	40.8 ± 13.3	42.3 ± 15.3	45.2 ± 13.1	40.8 ± 11.5	0.0001	**a > b (p = 0.0026)**	− 0.2 ± 0.2	− 0.2 ± 0.3	− 0.1 ± 0.3	− 0.2 ± 0.2	− 11.8 ± 11.2	− 10.2 ± 13.3	− 6.7 ± 16.9	− 11.7 ± 10.0
							**a > c (p = 0.0107)**								
							a > d (p = 0.1981)								
							**a > e (p = 0.0026)**								
*p* value (inter-group)	0.2744	0.8560	0.9035	0.5170	0.8703							0.6122	0.5741	0.8760	0.0990
*PI (%)*															
Probiotic group (n = 16)	54.6 ± 18.8	24.7 ± 11.3	25.2 ± 13.1	28.1 ± 14.6	25.1 ± 12.8	0.0003	**a > b (p = 0.0004)**	− 0.5 ± 0.3	− 0.5 ± 0.4	− 0.5 ± 0.3	− 0.5 ± 0.4	− 29.9 ± 17.2	− 29.3 ± 20.4	− 26.5 ± 19.4	− 29.4 ± 21.2
							**a > c (p = 0.0004)**								
							**a > d (p = 0.0006)**								
							**a > e (p = 0.0011)**								
Antibiotic group (n = 16)	58.6 ± 18.8	25.2 ± 14.0	32.6 ± 15.7	31.8 ± 14.8	32.7 ± 14.0	0.0001	**a > b (p = 0.0007)**	− 0.6 ± 0.3	− 0.5 ± 0.3	− 0.5 ± 0.3	− 0.5 ± 0.3	− 32.6 ± 16.5	− 26.0 ± 15.0	− 26.9 ± 14.4	− 26.0 ± 16.3
							**a > c (p = 0.0005)**								
							**a > d (p = 0.0004)**								
							**a > e (p = 0.0008)**								
Placebo group (n = 15)	56.1 ± 9.4	32.4 ± 13.9	27.6 ± 12.5	26.8 ± 13.3	35.8 ± 18.3	0.0030	**a > b (p = 0.0012)**	− 0.4 ± 0.3	− 0.5 ± 0.2	− 0.5 ± 0.3	− 0.4 ± 0.3	− 23.7 ± 15.7	− 28.9 ± 13.9	− 29.1 ± 15.3	− 20.3 ± 16.6
							**a > c (p = 0.0015)**								
							**a > d (p = 0.0010)**								
							**a > e (p = 0.0018)**								
*p* value (inter-group)	0.7774	0.2022	0.4261	0.6122	0.1789							0.3197	0.8377	0.8951	0.3817

Data presented as mean ± SD or number (%). *CAL* clinical attachment loss, *PPD* probing pocket depth, *BOP* bleeding on probing, *PI* Plaque index

^f^Effect size of (∆3 months‐BL) was calculated by delta mean change from month 3 to baseline over standard deviation of baseline. ^g^Effect size of (∆6 months‐BL) was calculated by delta mean change from month 6 to baseline over standard deviation of baseline. ^h^Effect size of (∆9 months‐BL) was calculated by delta mean change from month 9 to baseline over standard deviation of baseline. ^i^Effect size of (∆12 months‐BL) was calculated by delta mean change from month 12 to baseline over standard deviation of baseline

Intra-group comparison by Friedman test (*p* < 0.05) and Bonferroni-corrected Wilcoxon signed rank test (*p* < 0.0125). Statistical significant in bold

Inter-group comparison by Fisher's exact test, ANOVA and Kruskal Wallis test (*p* < 0.05)

The mean CAL, PPD, BOP and PI values at baseline and at 3, 6, 9, and 12 months follow-up of all groups are presented in Table 1. All treatment groups showed a significant reduction of PPD and PI at all timepoints, inter-group comparison however revealed that there were no significant differences between the treatment modalities (*p* > 0.05) (Table 1). In terms of CAL, the probiotic group showed a significant reduction at 3- and 9-month follow-ups whereas the antibiotic group showed a significant reduction at 3- and 6-month follow-ups. The placebo group on the other hand, showed a significant reduction of CAL at all time points (*p* < 0.0125). With respect to BOP, the antibiotic and placebo groups showed a significant reduction at 3-, 6- and 12-month follow-ups (*p* < 0.0125) whereas the probiotic group did not exhibit any improvement at any time point (*p* > 0.05).

The magnitude of statistical changes (effect size) in all clinical parameters from baseline to 3-, 6-, 9- and 12-month follow-up was similar in all treatment modalities (Table 1). Furthermore, inter-group comparison revealed no significant differences in the changes (∆) of any clinical parameter between the treatment groups (*p* > 0.05).

Moderate pockets (PPD = 4–6 mm) in the antibiotic group revealed an increase in CAL at 3 months and a significant reduction of PPD at 12 months. Moderate pockets also showed a significant reduction in PPD in the placebo group at 3- and 12-month follow up in placebo group (Table 2. All, *p* < 0.025).

**Table 2:** Clinical attachment loss (CAL) and probing pocket depth (PPD) in moderate (4–6 mm) and deep sites (≥ 7 mm) at baseline, 3-months and 12-months follow up

	Baseline (a)	3 months (b)	12 months (c)	*p* value (intra-group)	Post-hoc (Bonferroni adjustment)	Effect size (∆3 months-BL)^a^	Effect size (∆12 months-BL)^b^	∆3 months-BL	∆12 months-BL
*Moderate sites (CAL)*									
Probiotic group (n = 16)	5.6 ± 0.6	6.0 ± 1.0	6.1 ± 1.2	0.0238	a > b (p = 0.1208)	0.4 ± 0.9	0.5 ± 0.9	0.4 ± 0.9	0.5 ± 0.9
					a > c (p = 0.0747)				
Antibiotic group (n = 16)	5.9 ± 1.0	6.4 ± 1.3	6.4 ± 1.4	0.0018	**a < b (p = 0.0171)**	− 0.04 ± 0.7	0.5 ± 1.0	0.7 ± 0.9	0.5 ± 1.0
					a > c (p = 0.0340)				
Placebo group (n = 15)	6.1 ± 1.3	6.0 ± 1.7	6.5 ± 1.6	0.0006	a > b (p = 0.7333)	0.7 ± 1.0	0.4 ± 1.0	− 0.04 ± 0.7	0.4 ± 1.0
					a > c (p = 0.1398)				
*p* value (inter-group)	0.4031	0.6160	0.7649					0.0857	0.9483
*Deep sites (CAL)*									
Probiotic group (n = 16)	8.4 ± 1.8	9.3 ± 2.0	10.8 ± 3.6	0.0498	a > b (p = 0.7127)	0.05 ± 0.9	0.8 ± 1.1	0.1 ± 1.7	1.6 ± 2.2
					a > c (p = 0.1756)				
Antibiotic group (n = 16)	9.5 ± 2.1	10.4 ± 2.4	11.2 ± 1.7	0.0498	a > b (p = 0.6858)	0.09 ± 0.6	0.2 ± 0.6	0.2 ± 1.1	0.4 ± 1.1
					a > c (p = 0.5930)				
Placebo group (n = 15)	9.5 ± 2.1	11.1 ± 2.0	10.6 ± 2.1	0.3386	a > b (p = 0.2489)	0.4 ± 1.5	0.4 ± 0.8	0.9 ± 2.9	0.8 ± 1.6
					a > c (p = 0.1763)				
*p* value (inter-group)	0.4837	0.3254	0.9420					0.8257	0.6535
*Moderate sites (PPD)*									
Probiotic group (n = 16)	4.5 ± 0.3	4.4 ± 0.5	4.3 ± 0.3	0.0006	a > b (p = 0.1788)	− 0.2 ± 1.9	− 0.8 ± 1.4	− 0.06 ± 0.5	− 0.2 ± 0.4
					a > c (p = 0.0464)				
Antibiotic group (n = 16)	4.5 ± 0.2	4.4 ± 0.3	4.3 ± 0.2	0.0040	a > b (p = 0.0287)	− 0.6 ± 0.8	− 0.8 ± 0.7	− 0.2 ± 0.2	− 0.2 ± 0.2
					**a > c (p = 0.0010)**				
Placebo group (n = 15)	4.6 ± 0.3	4.2 ± 0.2	4.2 ± 0.3	0.0280	**a > b (p = 0.0007)**	− 1.4 ± 0.9	− 1.3 ± 1.2	− 0.4 ± 0.2	− 0.4 ± 0.3
					**a > c (p = 0.0012)**				
*p* value (inter-group)	0.6098	0.3097	0.7395					0.4489	0.3449
*Deep sites (PPD)*									
Probiotic group (n = 16)	7.5 ± 1.0	7.4 ± 0.9	7.9 ± 1.2	0.1054	a > b (p = 0.0947)	− 0.4 ± 0.3	− 0.1 ± 0.3	− 0.4 ± 0.3	− 0.1 ± 0.3
					a > c (p = 0.5716)				
Antibiotic group (n = 16)	7.5 ± 0.7	7.7 ± 0.7	7.1 ± 0.2	0.1054	a > b (p = 0.6845)	0.1 ± 1.4	− 0.2 ± 0.1	0.08 ± 1.1	− 0.2 ± 0.08
					a > c (p = 0.1088)				
Placebo group (n = 15)	7.8 ± 0.7	7.6 ± 0.9	7.2 ± 0.2	0.6747	a > b (p = 0.3991)	− 0.6 ± 1.8	− 1.0 ± 1.0	− 0.4 ± 1.4	− 0.8 ± 0.8
					a > c (p = 0.0510)			0.7364	0.2588
*p* value (inter-group)	0.3767	0.2995	0.1862						

Data presented as mean ± SD; BL: Baseline. CAL: Clinical attachment loss. PPD: Probing pocket depth

^a^Effect size of (∆3 months‐BL) was calculated by delta mean change from month 3 to baseline over standard deviation of baseline. ^b^Effect size of (∆12 months‐BL) was calculated by delta mean change from month 12 to baseline over standard deviation of baseline

Intra-group comparison by Friedman test (p < 0.05) and Bonferroni-corrected Wilcoxon signed rank test (*p* < 0.025). Statistical significant in bold

Inter-group comparison by ANOVA and Kruskal Wallis test (*p* < 0.05)

At 12 months follow-up, 80.3%, 77.8% and 77.2% of the baseline moderate pockets (PPD = 4–6 mm) presented a PPD ≤ 3 mm in the probiotic, antibiotic and placebo group respectively, without significant differences between the groups (*p* > 0.05) (Table 3). With respect to the baseline deep pockets (PPD > 7 mm), 42.4%, 41.7% and 38.8% of these pockets presented a PPD ≤ 3 mm, and 47%, 50% and 45.9% presented PPD = 4–6 mm, without significant between the treatment groups (*p* > 0.05) (Table 3). Pocket closure was 78.1%, 76.7% and 74.4% in the probiotic, antibiotic and placebo group, without significant differences between the treatment modalities (*p* > 0.05) (Table 3).

**Table 3:** Follow-up of moderate (4–6 mm) and deep pockets (≥ 7 mm) detected at baseline and pocket closure at 3- and 12- months follow up

	3 months	12 months
*Follow-up of moderate pockets detected at baseline (%)*		
PPD ≤ 3 mm		
Probiotic group (n = 16)	68.5 ± 16.4	80.3 ± 18.3
Antibiotic group (n = 16)	74.0 ± 13.7	77.8 ± 18.9
Placebo group (n = 15)	77.0 ± 14.7	77.2 ± 14.5
*p* value (inter-group)	0.2877	0.6950
PPD 4–6 mm		
Probiotic group (n = 16)	30.3 ± 15.9	18.8 ± 17.6
Antibiotic group (n = 16)	25.6 ± 13.0	20.9 ± 17.1
Placebo group (n = 15)	21.5 ± 13.1	21.5 ± 13.4
*p* value (inter-group)	0.2391	0.7886
PPD ≥ 7 mm		
Probiotic group (n = 16)	1.2 ± 2.0	0.9 ± 2.3
Antibiotic group (n = 16)	0.4 ± 1.4	1.3 ± 2.6
Placebo group (n = 15)	1.5 ± 3.8	1.3 ± 3.8
*p* value (inter-group)	0.2861	0.8539
*Follow-up of deep pockets detected at baseline (%)*		
PPD ≤ 3 mm		
Probiotic group (n = 16)	16.5 ± 28.5	42.4 ± 41.3
Antibiotic group (n = 16)	33.0 ± 39.9	41.7 ± 43.9
Placebo group (n = 15)	43.3 ± 38.7	38.8 ± 31.5
*p* value (inter-group)	0.2087	0.9798
PPD 4–6 mm		
Probiotic group (n = 16)	73.4 ± 30.9	47.0 ± 43.6
Antibiotic group (n = 16)	50.2 ± 37.3	50.0 ± 41.6
Placebo group (n = 15)	38.1 ± 28.1	45.9 ± 28.6
*p* value (inter-group)	0.1019	0.9715
PPD ≥ 7 mm		
Probiotic group (n = 16)	10.1 ± 17.7	10.6 ± 18.2
Antibiotic group (n = 16)	16.8 ± 21.1	8.3 ± 18.0
Placebo group (n = 15)	18.6 ± 26.6	15.3 ± 16.3
*p* value (inter-group)	0.6924	0.3030
Pocket closure (%)		
Probiotic group (n = 16)	65.3 ± 17.9	78.1 ± 20.0
Antibiotic group (n = 16)	71.9 ± 14.4	76.7 ± 19.7
Placebo group (n = 15)	73.3 ± 18.1	74.4 ± 16.8
*p* value (inter-group)	0.3790	0.6660

Data presented as mean ± SD. *PPD* probing pocket depth

Inter-group comparison by ANOVA and Kruskal Wallis test (p < 0.05)

The number of teeth and sites with PPD ≥ 5, ≥ 6 and ≥ 7 mm were significantly reduced in all groups and without significant difference between the treatment modalities at 12 months follow-up (*p* > 0.05) (Table 4). While probiotics reduced the percentage of patients with PPD ≥ 5 mm and PPD ≥ 6 mm at 12 months, antibiotics reduced the percentage of patients with PPD ≥ 6 mm and PPD ≥ 7 mm (*p* < 0.025) (Additional file 1: Table S1).

**Table 4:** Teeth and sites with PPD ≥ 5, ≥ 6 and ≥ 7 mm at baseline, 3- and 6–12 months follow up

	Baseline (a)	3 months (b)	12 months (c)	*p* value (intra-group)	Post-hoc (Bonferroni adjustment)	Effect size (∆3 months-BL)^a^	Effect size (∆12 months-BL)^b^	∆3 months-BL	∆12 months-BL
*Teeth with PPD ≥ 5 mm (%)*									
Probiotic group (n = 16)	33.3 ± 25.3	13.9 ± 12.5	9.7 ± 10.5	0.0040	**a > b (p = 0.0009)**	− 0.7 ± 0.7	− 0.9 ± 0.8	− 19.4 ± 18.1	− 23.5 ± 21.6
					**a > c (p = 0.0008)**				
Antibiotic group (n = 16)	42.3 ± 24.1	12.7 ± 11.7	11.6 ± 11.6	0.0008	**a > b (p = 0.0007)**	− 1.1 ± 0.6	− 1.1 ± 0.7	− 28.5 ± 16.0	− 30.7 ± 18.9
					**a > c (p = 0.0005)**				
Placebo group (n = 15)	46.4 ± 32.6	15.0 ± 21.1	15.2 ± 19.1	0.0034	**a > b (p = 0.0007)**	− 1.2 ± 0.9	− 1.2 ± 0.9	− 31.4 ± 25.3	− 31.2 ± 23.8
					**a > c (p = 0.0008)**				
*p* value (inter-group)	0.4163	0.8307	0.8075					0.1978	0.4069
*Teeth with PPD ≥ 6 mm (%)*									
Probiotic group (n = 16)	15.9 ± 15.4	6.4 ± 7.3	4.9 ± 6.2	0.0184	**a > b (p = 0.0165)**	− 0.5 ± 0.7	− 0.6 ± 0.7	− 9.6 ± 12.6	− 11.1 ± 13.8
					**a > c (p = 0.0089)**				
Antibiotic group (n = 16)	16.7 ± 12.6	6.4 ± 8.2	5.5 ± 8.1	0.0014	**a > b (p = 0.0009)**	− 0.5 ± 0.5	− 0.6 ± 0.5	− 10.2 ± 8.8	− 11.2 ± 9.0
					**a > c (p = 0.0007)**				
Placebo group (n = 15)	26.6 ± 25.8	8.4 ± 13.7	8.5 ± 12.2	0.0054	**a > b (p = 0.0025)**	− 1.0 ± 0.9	− 1.0 ± 1.0	− 18.2 ± 7.6	− 18.2 ± 18.4
					**a > c (p = 0.0043)**				
*p* value (inter-group)	0.6056	0.9650	0.6213					0.3702	0.2921
*Teeth with PPD ≥ 7 mm (%)*									
Probiotic group (n = 16)	8.3 ± 11.0	2.3 ± 2.9	1.8 ± 2.6	0.0490	a > b (p = 0.0738)	− 0.4 ± 0.7	− 0.5 ± 0.7	− 6.0 ± 9.7	− 6.5 ± 9.2
					**a > c (p = 0.0145)**				
Antibiotic group (n = 16)	7.6 ± 7.6	3.0 ± 5.2	2.4 ± 4.7	0.0049	**a > b (p = 0.0015)**	− 0.4 ± 0.4	− 0.4 ± 0.5	− 5.1 ± 5.5	− 5.2 ± 7.1
					**a > c (p = 0.0077)**				
Placebo group (n = 15)	16.1 ± 19.7	5.7 ± 8.9	5.1 ± 6.9	0.0469	**a > b (p = 0.0122)**	− 0.7 ± 0.9	− 0.8 ± 1.1	− 10.4 ± 13.1	− 11.0 ± 14.7
					**a > c (p = 0.0143)**				
*p* value (inter-group)	0.6573	0.8204	0.3481					0.6055	0.8091
*Sites with PPD ≥ 5 mm (%)*									
Probiotic group (n = 16)	10.9 ± 10.2	3.4 ± 3.6	2.4 ± 2.9	0.0043	**a > b (p = 0.0013)**	− 0.6 ± 0.6	− 0.7 ± 0.7	− 7.6 ± 7.9	− 8.5 ± 9.1
					**a > c (p = 0.0011)**				
Antibiotic group (n = 16)	14.1 ± 8.9	3.6 ± 4.3	3.3 ± 4.0	0.0005	**a > b (p = 0.0007)**	− 0.8 ± 0.4	− 0.8 ± 0.5	− 10.2 ± 5.7	− 10.8 ± 6.6
					**a > c (p = 0.0005)**				
Placebo group (n = 15)	20.0 ± 18.6	4.8 ± 7.2	4.3 ± 6.1	0.0019	**a > b (p = 0.0007)**	− 1.1 ± 1.1	− 1.2 ± 1.1	− 15.2 ± 14.6	− 15.7 ± 14.9
					**a > c (p = 0.0007)**				
*p* value (inter-group)	0.3755	0.8940	0.8700					0.2328	0.3160
*Sites with PPD ≥ 6 mm (%)*									
Probiotic group (n = 16)	4.3 ± 4.7	1.4 ± 1.7	1.2 ± 2.0	0.0180	**a > b (p = 0.0215)**	− 0.4 ± 0.5	− 0.4 ± 0.5	− 2.9 ± 3.7	− 3.1 ± 4.1
					**a > c (p = 0.0076)**				
Antibiotic group (n = 16)	4.9 ± 4.4	1.6 ± 2.7	1.3 ± 2.1	0.0049	**a > b (p = 0.0009)**	− 0.4 ± 0.3	0.4 ± 0.4	− 3.5 ± 2.6	− 3.6 ± 3.6
					**a > c (p = 0.0017)**				
Placebo group (n = 15)	10.2 ± 11.3	2.2 ± 3.5	2.1 ± 3.1	0.0013	**a > b (p = 0.0030)**	− 1.0 ± 1.1	− 1.0 ± 1.2	− 8.0 ± 9.1	− 8.1 ± 9.3
					**a > c (p = 0.0011)**				
*p* value (inter-group)	0.4818	0.9371	0.6105					0.3637	0.4272
*Sites with PPD ≥ 7 mm (%)*									
Probiotic group (n = 16)	1.8 ± 2.6	0.6 ± 1.0	0.6 ± 1.4	0.0407	a > b (p = 0.0738)	− 0.3 ± 0.5	− 0.3 ± 0.5	− 1.2 ± 1.9	− 1.2 ± 1.9
					**a > c (p = 0.0195)**				
Antibiotic group (n = 16)	1.9 ± 2.1	0.6 ± 1.3	0.6 ± 1.2	0.0042	**a > b (p = 0.0016)**	− 0.4 ± 0.4	− 0.3 ± 0.4	− 1.4 ± 1.6	− 1.4 ± 1.7
					**a > c (p = 0.0043)**				
Placebo group (n = 15)	4.7 ± 6.8	1.4 ± 2.4	1.1 ± 1.8	0.0423	**a > b (p = 0.0198)**	− 0.8 ± 1.4	− 0.9 ± 1.5	− 3.3 ± 5.5	− 3.6 ± 6.0
					**a > c (p = 0.0169)**				
*p* value (inter-group)	0.5671	0.8492	0.4180					0.5396	0.7226

Note: Data presented as mean ± SD of percentages. *BL* baseline, *PPD* probing pocket depth

^a^Effect size of (∆3 months‐BL) was calculated by delta mean change from month 3 to baseline over standard deviation of baseline. ^b^Effect size of (∆12 months‐BL) was calculated by delta mean change from month 12 to baseline over standard deviation of baseline

Intra-group comparison by Friedman test (p < 0.05) and Bonferroni-corrected Wilcoxon signed rank test (p < 0.025). Statistical significant in bold

Inter-group comparison by ANOVA and Kruskal Wallis test (*p* < 0.05)

The use of antibiotics significantly increased the number of patients with low risk for disease progression at 12 months, and also significantly reduced the number of patients at higher risk for disease progression (Additional file 1: Table S1). In addition, the antibiotic and placebo group showed at 12 months a reduced necessity for additional therapy in ≥ 3 sites but without significant differences between the both groups (Additional file 1: Table S1).

## Discussion

This triple-blind placebo-controlled parallel-arm randomized clinical trial evaluated the clinical effects of *L. rhamnosus* SP1 administered once a day for 3 months or azithromycin plus SRP in stage III periodontitis generalized grade B. The present study predominantly revealed: (1) a significant improvement of PPD and PI irrespective of the treatment modality and without significant differences between the groups; (2) no added benefit of probiotics or azithromycin in terms of CAL; (3) a significant reduction in the number of sites and teeth with PPD ≥ 5, ≥ 6 and ≥ 7 mm in all groups at 12 month follow-up without differences between the treatment regimes. Therefore, the null hypothesis of the present study could not be rejected.

This is the first study evaluating and comparing the use of probiotics and antibiotics, in this case azithromycin, in the treatment of stage III periodontitis generalized grade B with 1-year follow-up. In general, all treatment groups revealed a significant improvement in PPD, BOP, CAL and PI, however, without significant differences between the treatment modalities. Hence, these results failed to exhibit an added benefit of probiotics and azithromycin. With respect to probiotics, the present results are in line with previous reports using probiotics as an adjunct therapy to SRP for the management periodontitis. The administration of *Lactobacillus reuteri* DSM-17938 + ATCCPTA 5289 [27], *Streptococcus oralis* KJ3 + *Streptococcu uberis* KJ2 + *Streptococcu rattus* JH145 [28] or *L. rhamnosus* SP1 [29] in conjunction with SRP did not enhance the clinical outcomes. Our observations are further supported by a very recent RCT which failed to demonstrate an additional clinical efficacy by the use of probiotics [30]. In that RCT, patients received *L. reuteri* ATCC PTA5289 twice a day in addition to SRP for 28 days and the patients were recalled at 3 and 6 months. The use of probiotics failed to provide a clinical benefit compared to SRP alone. Our study revealed a reduction in the percentage of patients, teeth and sites with PPD ≥ 5 mm, ≥ 6 mm and ≥ 7 mm in all groups, however without inter-group differences. These observations are supported by previous reports indicating a lack of effect of probiotics [29, 30]. Overall, these findings, in addition to the results of the present study, suggest that probiotics may not provide an added benefit to SRP at least in this type of patient.

Our findings, on the other hand are in contrast to previous reports showing an added benefit of probiotics. Patients that received *L. reuteri* DSM-17938 + ATCCPTA 5289 in conjunction to SRP showed a significantly higher PPD reduction along with a higher CAL gain at 12 month follow-up. Moreover, the probiotic group revealed a higher reduction of sites and patients with ≥ 6 mm [31] which could not be found in the present study. Outcomes from another report showed that patients who received *L. reuteri* ATCC 55,730 + ATCCPTA 5289 exhibited a higher reduction of CAL, PPD and BOP [32]. Similarly, the intake for 8 weeks of *Lactobacillus salivarius* WB21 by patients with periodontitis significantly enhanced the reduction of PPD at two month follow-up [33]. Furthermore, outcomes from a more recent RCT revealed a higher reduction of PPD and BOP by the use of *L. reuteri* lozenges [34]. These positive results were corroborated by another group using *Bifidobacterium animalis* subsp. *lactis* HN019-showing improved PPD and CAL at 3 month follow-up [35]. This notable discrepancy with our findings might be attributed to the probiotic itself [36]. One might speculate that not all probiotics produce the same effect, particularly in addition to SRP. Moreover, other factors such as strain, concentration, vehicle and administration time of the probiotic might account for these divergent results. Indeed, the selection of the ‘‘best’’ probiotic for oral health is still a matter of debate. The selection of *L. rhamnosus* SP1 for the present study was based on the beneficial effects in the immune response of children and adults [37–39]. Presumably, this immune modulation elicited by the Lactobacillus strain might limit the detrimental immune response observed in periodontitis.

As for the use of azithromycin, the present results in general failed to show a clear clinical advantage over SRP and placebo. These findings are in accordance with previously published data where a clinical benefit of azithromycin in conjunction with SRP could not be determined [22, 40, 41]. This lack of additional benefit of azithromycin is in direct contrast with the conclusions of a recent systematic review with meta-analysis [42]. These differences might be attributed to the higher potential of meta-analysis to estimate an overall mean effect. Indeed, we found a significant reduction in the number of patients with PPD ≥ 7 mm in the azithromycin group, however this difference was not robust enough to show a significant benefit compared to the other groups. It has been suggested that azithromycin produces some minor benefits. In this regard, it should be noted that the inclusion of 3 treatment groups along with the corresponding correction for multiple comparison may have influenced the power to find minor differences between the groups. Thus, azithromycin may produce some benefits but they could not be detected by our clinical trial. A recent systematic review underlined that the reported clinical advantage of azithromycin should be interpreted with caution since the major effect was derived from a single study [42] with a high risk of bias.

We recognize that this study has a number of limitations. First, the small sample size. Although a sample size calculation was performed, a larger sample size would allow for the detection of smaller differences between the adjunctive therapeutic agents. However, it should be noted that there is no consensus from which point a statistical difference is clinically relevant. Second, microbiological and samples were not taken, therefore it is unclear whether the probiotics triggered a microbial shift. In this sense, whether the probiotic bacteria actually colonized the oral cavity remains to be elucidated. Third, since the present study only included patients with stage III periodontitis grade B, the effect of probiotics or azithromycin on other stages and grades of periodontitis has yet to be determined.Future studies should include larger populations in different stages and grades of periodontitis.

## Conclusion

In conclusion and within the limitations of the present study, the administration of *L. rhamnosus* SP1 or azithromycin in the treatment of stage III periodontitis generalized grade B failed to produce additional beneficial effects when compared to SRP on its own. Given the lack of benefits and the conflicting data in the literature, the benefits of probiotics and azithromycin as an adjunct to SRP in the treatment of periodontitis remains unclear.

## Supplementary information

**Additional file 1:** Patients with PPD ≥5, ≥6 and ≥7mm, in risk of progression and in need for additional therapy at baseline, 3- and 12-months follow up.

## Data Availability

The datasets used and/or analysed during the current study are available from the corresponding authors on reasonable request.

## Abbreviations

BOP: Bleeding on probing
CAL: Clinical attachment loss
PPD: Probing pocket depth
SD: Standard deviation
SRP: Scaling and root planing
PI: Plaque accumulation
WHO: World Health Organization
